# Surfaceome Reprogramming of Stemsomes Promotes Lung Cancer Targeting via Potentiated Receptor–Ligand Interactions

**DOI:** 10.1002/advs.76039

**Published:** 2026-06-09

**Authors:** Geunhye Kim, In Ah Kwon, Bo Seop Jeong, Hyo Seon Kim, Joo‐Hwan Park, Sung Jean Park, Sang‐Rae Lee, Youngki Lee, Dongwoo Khang

**Affiliations:** ^1^ Department of Health Sciences and Technology GAIHST Gachon University Incheon South Korea; ^2^ Ectosome Inc. Incheon South Korea; ^3^ Department of Physiology College of Medicine Gachon University Incheon South Korea; ^4^ Division of Medical Oncology Department of Internal Medicine Gachon University School of Medicine Gil Medical Center Incheon South Korea; ^5^ College of Pharmacy and Gachon Institute of Pharmaceutical Sciences Gachon University Incheon South Korea; ^6^ Department of Pharmacology Ajou University School of Medicine Suwon South Korea; ^7^ Efficacy Test Center For Mental & Behavioral Disorders Ajou University Hospital Suwon South Korea; ^8^ Department of Biopharmaceutical Sciences Cheongju University Cheongju South Korea; ^9^ Lee Gil Ya Cancer and Diabetes Institute Gachon University Incheon South Korea

**Keywords:** cell membrane‐derived vesicle, dexamethasone, liposome, lung cancer, mesenchymal stem cell

## Abstract

Non‐small‐cell lung cancer (NSCLC) mortality remains high because mutation‐specific therapies target only small patient subsets and inevitably encounter drug resistance. In this regard, multipathway‐assisted mesenchymal stem cell‐derived nanovesicles (stemsomes) offer a promising universal platform for overcoming the unmet needs of lung cancer therapeutics, particularly for patients lacking identifiable oncogenic drivers. This study introduces a novel strategy in which dexamethasone, a compound conventionally used as an anti‐inflammatory drug, is repurposed to enhance the tumor‐targeting capabilities of stemsomes. Nanoparticles engineered by fusing these dexamethasone‐primed stemsomes with liposomes exhibit markedly improved therapeutic effects. According to transcriptomic and siRNA‐mediated knockdown experiments, this enhanced targeting of tumor cells is driven by the dexamethasone‐induced upregulation of key cell adhesion proteins, specifically ephrin type‐A receptor 2 and neurogenic locus notch homolog protein 3. Furthermore, a comprehensive map of potential adhesion interactions and computational simulations suggest a multivalent interaction network between the surfaces of dexamethasone‐primed stemsomes and NSCLC H1975 cells. These findings indicate the discovery of a highly translational and mutation‐independent strategy that represents a promising novel mechanism for engineering vesicle surfaces for NSCLC therapy.

## Graphical abstract

This study presents a surfaceome‐reprogramming strategy for mutation‐independent lung cancer therapy by repurposing dexamethasone to prime mesenchymal stem cell‐derived nanovesicles. The engineered vesicles leverage multi‐valent interactions mediated by upregulated adhesion proteins, EPHA2, and NOTCH3. This platform provides a scalable and translational mechanism for enhancing tumor‐targeting efficiency, offering a new paradigm for developing cell‐inspired nanomedicines.

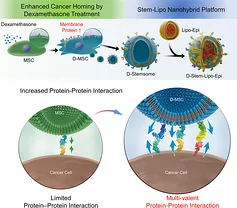

## 1 Introduction

Lung cancer remains the most lethal malignancy globally and is responsible for approximately 1.8 million deaths annually, with non‐small‐cell lung cancer (NSCLC) constituting over 85% of cases [1]. The effective management of advanced‐stage NSCLC is hindered by limited tumor specificity and considerable systemic toxicity associated with traditional chemotherapy [2, 3, 4]. While various targeted therapies were developed, their clinical application remains restricted to specific patient subsets. For instance, epidermal growth factor receptor (EGFR) mutations and anaplastic lymphoma kinase rearrangements represent approximately 20% and 3% of cases, respectively [5]. However, 38% of patients with NSCLC lack identifiable oncogenic drivers, which excludes them from existing targeted therapeutic options. Furthermore, inevitable drug resistance leaves the vast majority of the population without effective long‐term treatment. In 50% of cases, drug resistance is driven by amplification of the mesenchymal–epithelial transition factor, and 15% of patients treated with third‐generation tyrosine kinase inhibitors like osimertinib also undergo histological transformation to more aggressive cancer types [6]. This therapeutic gap underscores a critical unmet need for universal strategies that can overcome the limitations through multi‐pathway approaches.

To address these challenges, lipid‐based nanoparticles have gained attention due to their biocompatibility and capacity to encapsulate various drugs [7, 8, 9]. However, conventional liposomes lack intrinsic targeting mechanisms, which often leads to their rapid clearance by the reticuloendothelial system and inadequate tumor selectivity [10, 11]. In this study, the hybridization of liposomes with cell‐derived membranes was introduced, which has emerged as a promising biomimetic strategy [12, 13]. Membranes derived from mesenchymal stem cells (MSCs) are particularly attractive, as MSCs possess an inherent ability to migrate toward tumor environments and inflammatory sites [14, 15]. This well‐known tropism is guided by specific chemokine receptors, such as C‐X‐C chemokine receptor type 4 (CXCR4) and C─C chemokine receptor type 2, and the engagement of adhesion molecules, including the integrin very late antigen‐4 [16, 17].

While promising, the direct clinical use of live MSCs is hampered by critical safety concerns, like their potential tumorigenicity, leading to the emergence of MSC‐derived extracellular vesicles (stemsomes) as a compelling cell‐free alternative [18, 19, 20]. These vesicles retain the tumor‐targeting properties of their parent MSCs without the associated cellular risks [21, 22, 23]. Despite these advantages, these cell‐free vesicles lack the active migration machinery of their parent cells. Consequently, considerable research has focused on surface engineering strategies that involve the conjugation of specific targeting moieties, such as ligands or antibodies [24, 25].

Instead of these commonly employed direct surface modifications, the current study explored chemical preconditioning of MSCs as a strategy to produce stemsomes with amplified targeting moieties on their surfaces. In this context, the glucocorticoid dexamethasone has been investigated as a potential preconditioning agent. Dexamethasone is widely used as an anti‐inflammatory drug and osteogenic differentiation agent for MSCs [26, 27, 28]. However, its effects on cell migration are complex and context‐dependent. At low concentrations, glucocorticoids can promote MSC migration by increasing the expression of N‐formyl peptide receptors while decreasing the expression of CXCR4, which facilitates homing to sites of injury [29]. Conversely, at higher pharmacological concentrations, dexamethasone substantially impairs MSC migration through mechanisms involving cytoskeletal rigidity [30, 31]. Furthermore, dexamethasone broadly modulates cell adhesion by altering the expression profile of key surface molecules, including integrin alpha 5 and various cadherins crucial for cell–cell interactions [32, 33, 34]. In addition, some studies suggested that these steroids can promote cancer metastasis and migration [35, 36]. Therefore, this study hypothesized that such remodeling capabilities of dexamethasone could be repurposed to artificially enhance the targeting profile of MSC‐derived nanovesicles.

Based on this background, the enhanced tumor‐homing ability of parent MSCs following dexamethasone treatment (D‐MSC) was confirmed. Subsequently, stemsomes derived from dexamethasone‐primed MSCs (D‐Stemsome) were hybridized with epirubicin‐encapsulating liposomes (Lipo‐Epi) to prepare D‐Stem‐Lipo‐Epi nanoparticles. The superior targeting capability induced by dexamethasone preconditioning was maintained in these nanoparticles, as demonstrated by the enhanced tumor accumulation and cellular uptake in models of NSCLC, both in vitro and in vivo. Mechanistic studies revealed that this enhanced targeting can be attributed to the upregulation of cell‐binding molecules such as ephrin type‐A receptor 2 (EPHA2) and neurogenic locus notch homolog protein 3 (NOTCH3). Furthermore, transcriptomic analyses and computational simulations identified multiple potential protein–protein interactions (PPIs) that constitute a multivalent binding network.

Consequently, novel, chemically primed, biomimetic nanoparticles were developed in this study, and their therapeutic potential for NSCLC was elucidated. This study not only establishes that the targeting efficacy of MSC‐derived vesicles can be augmented by repurposing conventional glucocorticoids but also identifies several cell adhesion molecules as key mechanistic drivers for this enhancement.

## 2 Results

### 2.1 Dexamethasone Preconditioning Enhances the Lung Tumor‐Homing Ability of MSCs

**SCHEME 1:**
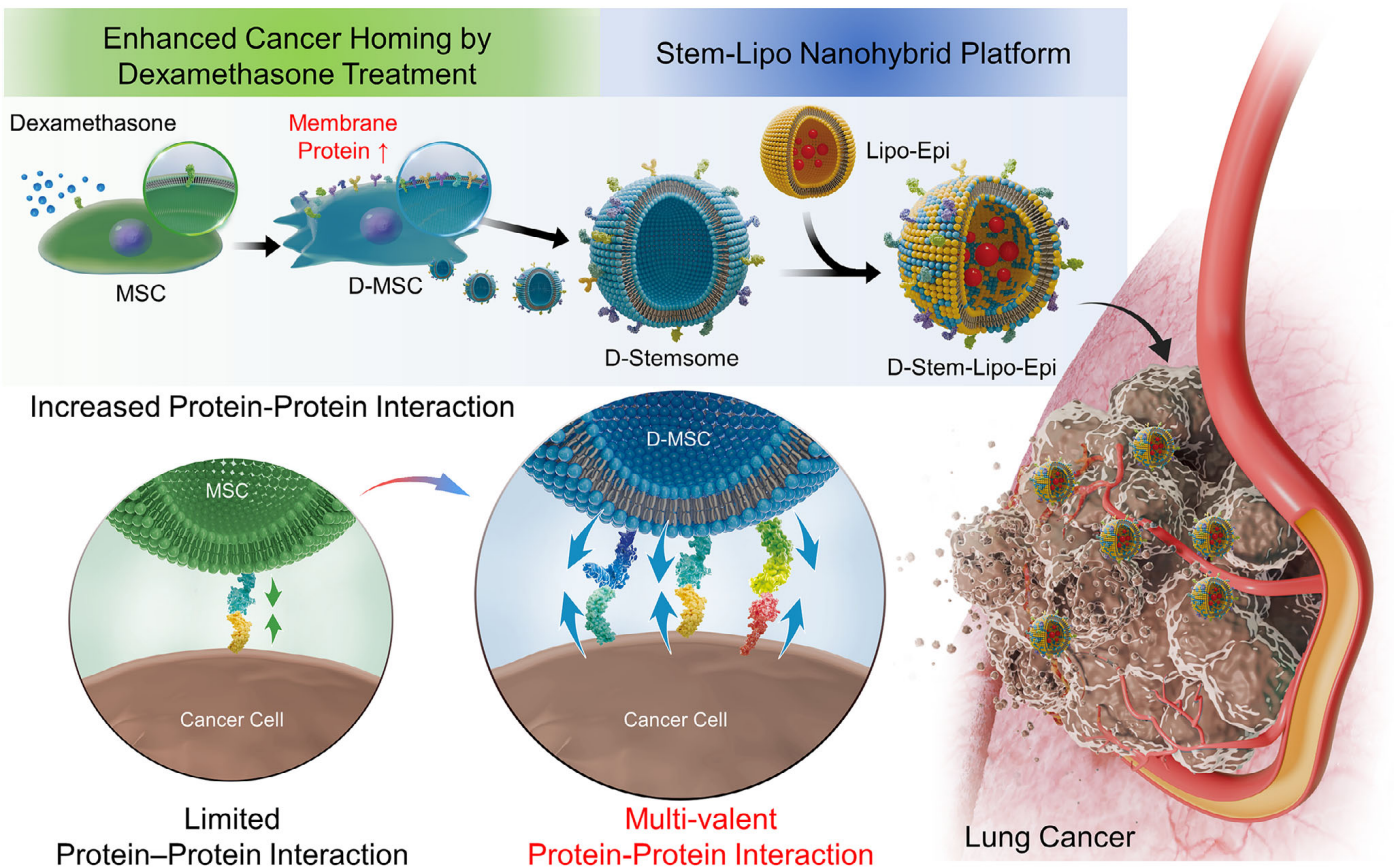
Surfaceome engineering of the stemsome platform with dexamethasone for targeted lung cancer therapy. Stemsome–Liposomes are generated by fusing epirubicin‐loaded liposomes with nanovesicles derived from dexamethasone‐primed mesenchymal stem cells. The resulting nanoparticles specifically target lung cancer cells through multivalent protein–protein interactions, facilitating drug delivery and effective tumor eradication.

**FIGURE 1:**
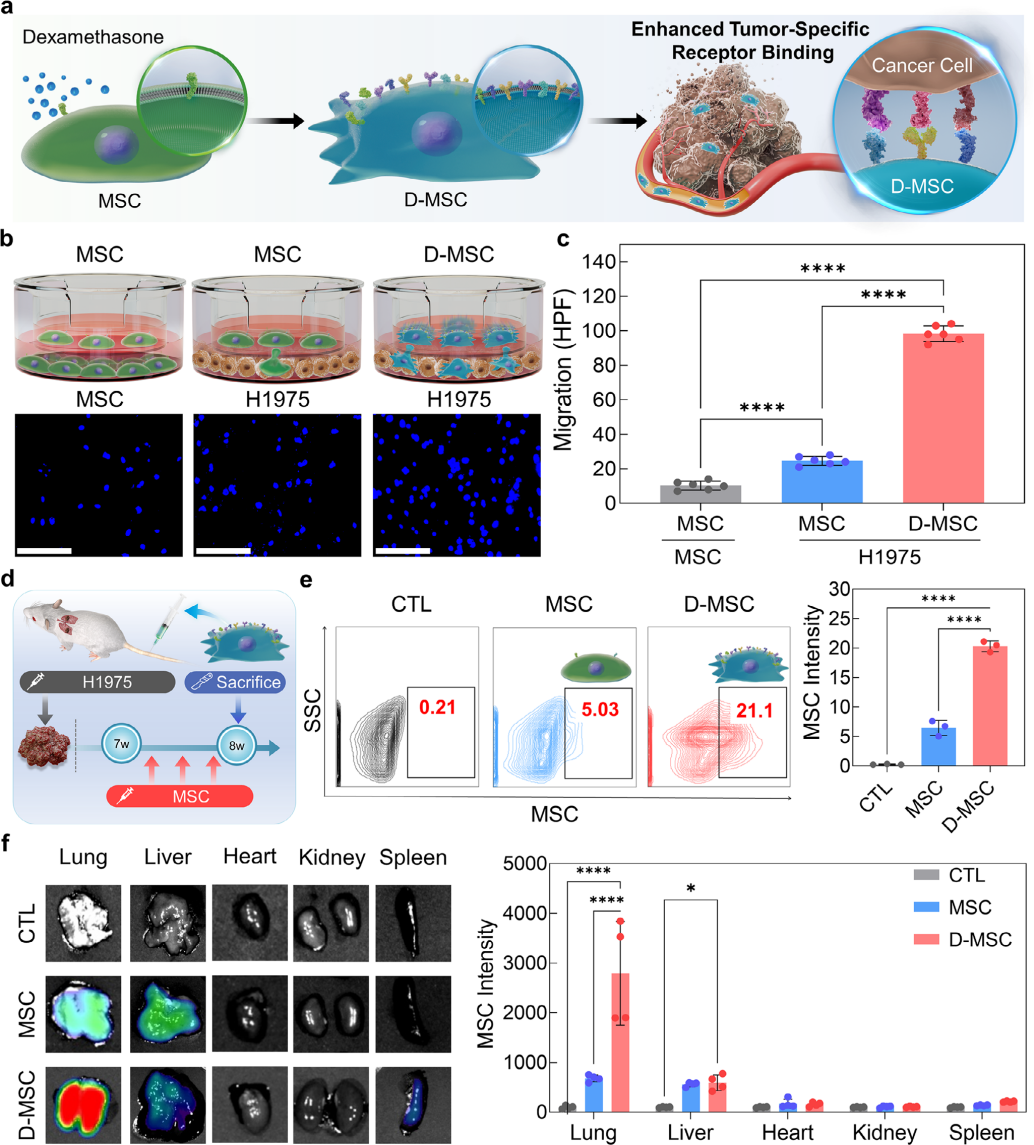
Enhanced homing ability of dexamethasone‐primed mesenchymal stem cells (D‐MSCs). (a) Schematic illustration of dexamethasone treatment of mesenchymal stem cells (MSCs) to generate D‐MSCs with enhanced tumor‐specific binding. (b) Representative images of an in vitro transwell migration assay showing increased nuclei of migrated MSCs stained with DAPI (blue). Scale bars, 50 µm. (c) Quantification of migrated MSCs per HPF. Data are presented as mean ± SD (*n* = 6). ^****^ *p* < 0.0001. (d) Schematic illustration of the in vivo tumor‐homing evaluation in a mouse model of non‐small‐cell lung cancer (NSCLC). (e) Representative flow cytometry plots and MSC quantification within tumors. Data are presented as mean ± SD (*n =* 3). ^****^ *p* < 0.0001. (f) Ex vivo fluorescence images and quantification of labeled MSCs in major organs. Data are presented as mean ± SD (*n =* 4). ^*^ *p* < 0.05, ^****^ *p* < 0.0001. CTL, control; HPF, high‐power field.

The overall strategy for dexamethasone‐induced surface reprogramming of MSCs and the subsequent engineering of multivalent stemsomes is illustrated in Scheme 1. Building on the hypothesis that conventional glucocorticosteroids can reprogram cellular migration profiles, a dexamethasone‐based preconditioning strategy was developed to optimize the homing efficiency of MSCs (Figure 1a). To validate this preconditioning strategy, the effects of dexamethasone priming on MSCs were evaluated using an in vitro transwell migration assay (Figure 1b,c). The dexamethasone concentration was determined based on a dose optimization study (Figure S1). While naïve MSCs exhibited a significant migratory capacity toward the NSCLC cell line H1975, this effect was significantly enhanced when MSCs were pretreated with dexamethasone. The number of migrated D‐MSCs was fourfold greater than that of the naïve MSC group, suggesting improved tumor‐homing ability after dexamethasone treatment.

Based on these promising in vitro results, the enhanced tumor‐homing effect was validated using an animal model. An orthotopic NSCLC mouse model was established using H1975 cells, and fluorescently labeled naïve MSCs or D‐MSCs were administered intravenously (Figure 1d). To quantify the accumulation of MSCs within the tumors, the harvested tumors were analyzed by flow cytometry. The analysis revealed that the population of D‐MSCs within the tumor was 3.1‐fold greater than that of the naïve MSC group, confirming the enhanced tumor‐homing efficacy of dexamethasone priming (Figure 1e). The targeting ability of the D‐MSCs was further corroborated by ex vivo biodistribution imaging of major organs (Figure 1f). D‐MSCs demonstrated a 4.1‐fold higher accumulation in tumor‐bearing lungs than naïve MSCs. Notably, only moderate differences were observed in off‐target organs, such as the liver and kidney, highlighting the specificity of the targeting by MSCs. Collectively, these results demonstrate that dexamethasone preconditioning improves the tumor homing of MSCs to lung cancers.

### 2.2 Preparation and Characterization of Stemsome‐Liposome Platform

**FIGURE 2:**
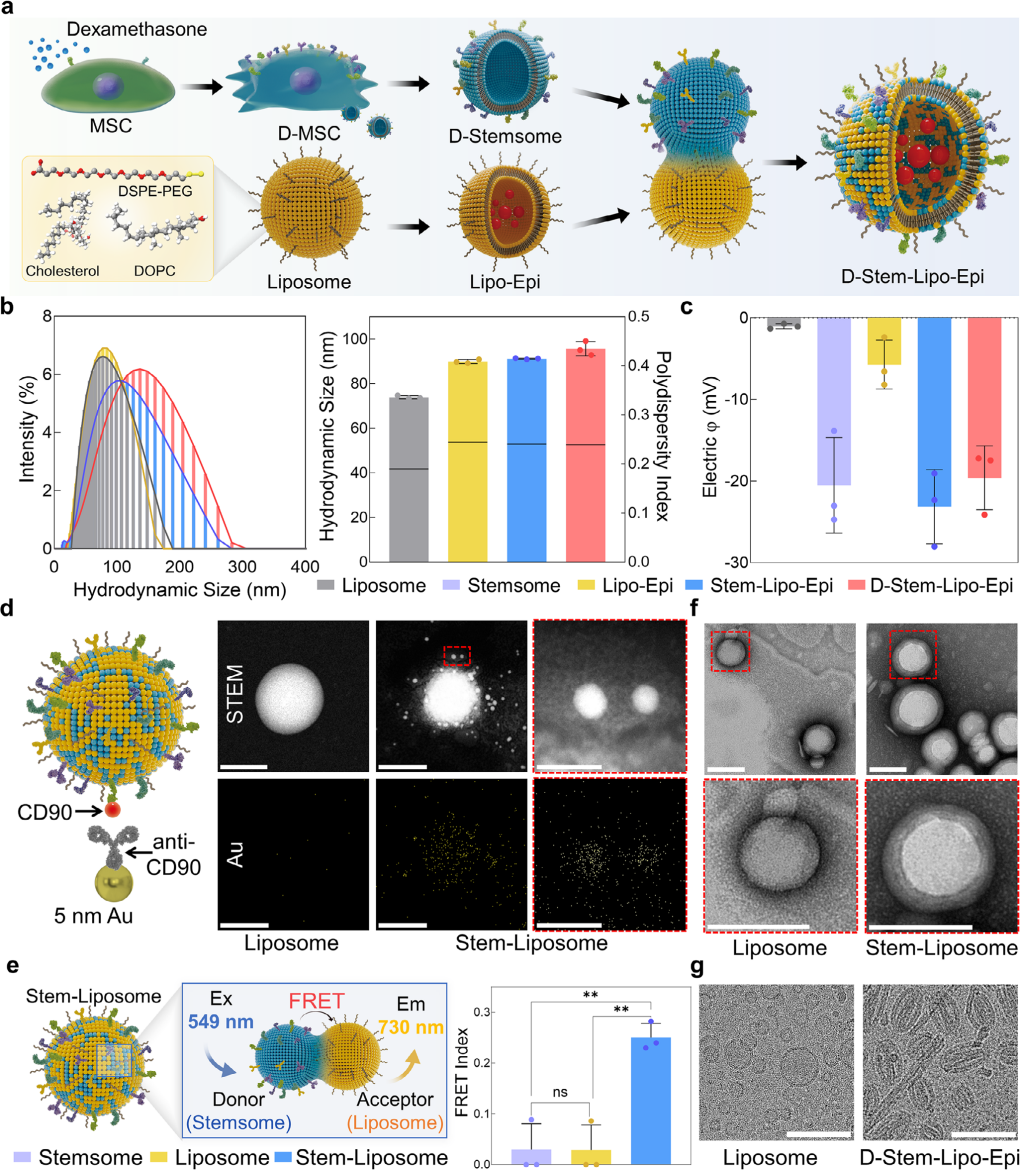
Preparation and physicochemical characterization of D‐Stem‐Lipo‐Epi. (a) Schematic illustration of the fabrication process. D‐MSCs were used to generate stemsomes, which were subsequently fused with epirubicin‐loaded liposomes (Lipo‐Epi) to form D‐Stem‐Lipo‐Epi. (b) Scatter and bar plots represent the hydrodynamic diameter (left axis), and internal horizontal lines denote the polydispersity index (right axis). (c) Zeta potential of the indicated nanoparticles. Data are presented as mean ± SD (*n =* 3). (d) Scanning transmission electron microscopy and x‐ray spectroscopy images showing the gold (Au) nanoparticle labeling of CD90 proteins. Scale bars, 100 nm (main) and 20 nm (inset). (e) Förster resonance energy transfer assay demonstrating membrane fusion between donor stemsomes (excitation at 549 nm) and acceptor liposomes (emission at 730 nm). Data are presented as mean ± SD (*n =* 3). ^**^ *p* < 0.01; ns, not significant. (f) Transmission electron microscopy images of liposomes and Stem‐Liposomes. Scale bar, 100 nm. (g) Cryogenic transmission electron microscopy images of liposomes and D‐Stem‐Lipo‐Epi. Scale bar, 50 nm.

Despite the natural ability to target tumors, the clinical application of MSCs is restricted by their inherent tumorigenicity [19, 37]. To address this critical limitation, this study produced stemsomes through the ectosome extraction method [38]. Unlike exosomes, this approach retains membrane proteins of parental cells that can inherit advantageous targeting characteristics of MSCs while eliminating their tumorigenic risk [39]. To develop these advanced delivery vehicles, nanoparticles were designed by fusing stemsomes with liposomes, and the successful transfer of parental proteins to the nanoparticles was confirmed by sodium dodecyl sulfate‐polyacrylamide gel electrophoresis (SDS‐PAGE) (Figure S1). Lipo‐Epi was prepared using a remote loading method based on a pH gradient, similar to clinically established formulations such as Doxil, to ensure stable drug encapsulation (Figure 2a) [40, 41]. Dynamic light scattering analysis revealed that Lipo‐Epi had a uniform size distribution, with a mean diameter of 89.9 ± 0.5 nm and a stable polydispersity index of approximately 0.25 (Figure 2b). Following hybridization with stemsome membranes, the mean diameter of the resulting D‐Stem‐Lipo‐Epi increased slightly to 95.6 ± 1.8 nm. In addition, the zeta potential shifted from approximately −5 mV for Lipo‐Epi to approximately −20 mV for D‐Stem‐Lipo‐Epi (Figure 2c). This value became close to those of cell membranes and stemsomes, indicating the successful fusion of the nanoparticles. To further confirm the successful integration of stemsomes and liposomes, two additional experiments were performed. First, immuno‐gold labeling targeting the MSC‐specific surface marker CD90 was conducted as previously described [42]. Subsequently, scanning transmission electron microscopy (STEM) coupled with energy‐dispersive x‐ray spectroscopy (EDS) was performed. The analysis detected gold signals on the surface of stemsome–liposomes (Stem–Lipo), but not on control liposomes, indicating the successful transfer of membrane proteins from MSCs (Figure 2d). In addition, expression of CD44 was confirmed by western blot and flow cytometry (Figure S1). Finally, Förster resonance energy transfer (FRET) experiments were performed using a donor–acceptor fluorophore pair on liposomes and stemsomes, respectively [43]. The result provided definitive evidence of membrane fusion by detecting a strong FRET signal only upon successful hybridization (Figure 2e). Transmission electron microscopy (TEM) imaging demonstrated the uniform and spherical morphology of the resulting nanoparticles (Figure 2f). Successful drug loading was confirmed by cryogenic TEM, which revealed rod‐shaped epirubicin crystals inside the vesicles, a hallmark of the pH gradient loading method (Figure 2g) [40, 44]. In addition, drug content and encapsulation efficiency of Lipo‐Epi and Stem‐Lipo‐Epi were evaluated, which resulted in 59.02% ± 2.02% and 35.09% ± 0.97%, respectively. Furthermore, the stability of D‐Stem‐Lipo‐Epi was evaluated by monitoring its hydrodynamic size under various pH and 10% fetal bovine serum (FBS) conditions (Figure S1). The results showed that our Stem–Lipo system maintains structural integrity in various physiological conditions. Collectively, these data validate the successful fusion of the two components and confirm the establishment of a Stem‐Lipo nanoplatform.

### 2.3 Enhanced In Vitro Cellular Uptake and Cytotoxicity

**FIGURE 3:**
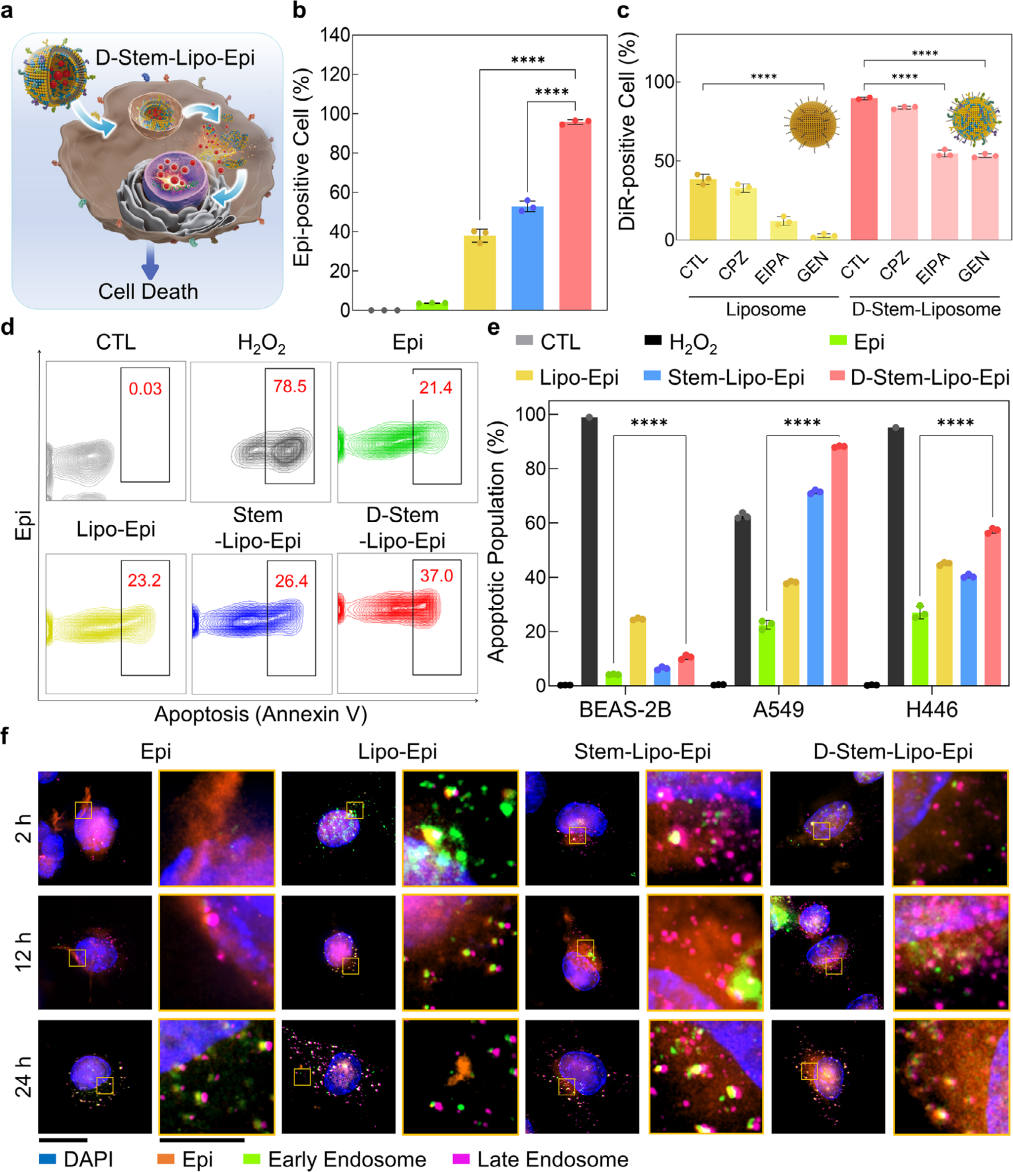
Cellular uptake, apoptosis, and intracellular trafficking of D‐Stem‐Lipo‐Epi. (a) Schematic illustration of the intracellular trafficking of D‐Stem‐Lipo‐Epi. (b) Flow cytometric analysis showing their intracellular uptake in the H1975 cell line. Data are presented as mean ± SD (*n =* 3). ^****^ *p* < 0.0001. (c) Cellular uptake of Epi in the presence of the endocytosis inhibitors chlorpromazine (CPZ, clathrin‐mediated), 5‐(N‐ethyl‐N‐isopropyl) amiloride (EIPA, macropinocytosis), and genistein (GEN, caveolae‐mediated). Data are presented as mean ± SD (*n =* 3). ^****^ *p* < 0.0001. (d, e) Apoptosis assays in (d) H1975 (NSCLC) and in (e) A549 (NSCLC), H446 (small cell lung cancer), and BEAS‐2B (normal bronchial epithelial) cells. Data are presented as mean ± SD (*n =* 3). ^****^ *p* < 0.0001. (f) Representative confocal images at 2, 12, and 24 h. Nuclei (blue), Epi (orange), early endosome marker (green), and late endosome/lysosome marker (magenta) are stained. Scale bars, 10 µm (main) and 5 µm (inset). CTL, control; Epi, epirubicin.

The therapeutic efficacy of the nanoparticles was evaluated at the cellular level (Figure 3a). The internalization of nanoparticles into H1975 NSCLC cells was quantified using flow cytometry. The D‐Stem‐Lipo‐Epi group exhibited the highest cellular uptake among all tested formulations (Figure 3b). Notably, its uptake was significantly greater than that of the Stem‐Lipo‐Epi group, confirming that dexamethasone preconditioning can enhance tumor cell‐targeting ability. The internalization pathway was subsequently evaluated by assessing the cellular uptake in the presence of various endocytosis inhibitors. Cells were pretreated with the inhibitors chlorpromazine, genistein, and 5‐(N‐ethyl‐N‐isopropyl)amiloride (EIPA) [45]. The cellular uptake of Lipo‐Epi and D‐Stem‐Lipo‐Epi was significantly inhibited by genistein and EIPA (Figure 3c). This indicates that their internalization is not dependent on a single route, but rather involves a combination of caveolae‐mediated endocytosis and macropinocytosis [46]. Crucially, fusion with the stemsome membrane did not appear to fundamentally alter the internalization pathways, suggesting that biomimetic coating enhances tumor‐cell affinity without restricting the modes of cellular entry.

Subsequently, the anticancer effects of the nanoparticles were evaluated. Treatment with D‐Stem‐Lipo‐Epi demonstrated significantly higher cytotoxicity in H1975 than any other treatment, which was not attributed to vehicle toxicity (Figure S1). In addition, flow cytometry analysis was performed to quantify apoptotic cell populations and intracellular epirubicin accumulation (Figure 3d). Consistent with the viability data, the D‐Stem‐Lipo‐Epi group exhibited the highest apoptotic signals, validating the potent therapeutic efficacy of these nanoparticles in vitro. Furthermore, the versatility and tumor specificity were evaluated using additional lung cancer cell lines (A549 and H446) and the immortalized normal lung epithelial cells BEAS‐2B (Figure 3e). D‐Stem‐Lipo‐Epi consistently induced significant apoptosis in the lung cancer cell lines but had minimal effects on non‐cancerous BEAS‐2B cells. These findings suggest that the Stem‐Lipo platform could serve as a universal therapeutic strategy for lung cancer that is effective across different histological types, while minimizing off‐target effects on normal lung tissue.

To understand the mechanism driving this superior cytotoxicity, the intracellular fate of the nanoparticles, which is particularly critical for epirubicin, was assessed. As an anthracycline, epirubicin exerts its cytotoxic effect by intercalating with DNA in the cell nucleus [47]. Therefore, efficient delivery to the nucleus is crucial for its therapeutic effect [48]. Fluorescence microscopy revealed that free epirubicin remained largely within the cytosol (Figure 3f). In contrast, Lipo‐Epi was initially localized to early endosomes and was observed after 12 h in both the cytosol and nucleus. Therefore, the encapsulation of epirubicin offers a clear advantage over the delivery of free epirubicin. Both Stem‐Lipo‐Epi and D‐Stem‐Lipo‐Epi were predominantly localized in the early and late endosomes. Although both fusion groups showed greater nuclear accumulation, the D‐Stem‐Lipo‐Epi group showed markedly higher epirubicin localization within the nucleus. Taken together, these results demonstrate that the D‐Stem‐Lipo‐Epi approach is a highly effective strategy that leverages enhanced cellular uptake and intracellular trafficking to achieve potent anticancer outcomes.

### 2.4 In Vivo Therapeutic Efficacy of D‐Stem‐Lipo‐Epi

**FIGURE 4:**
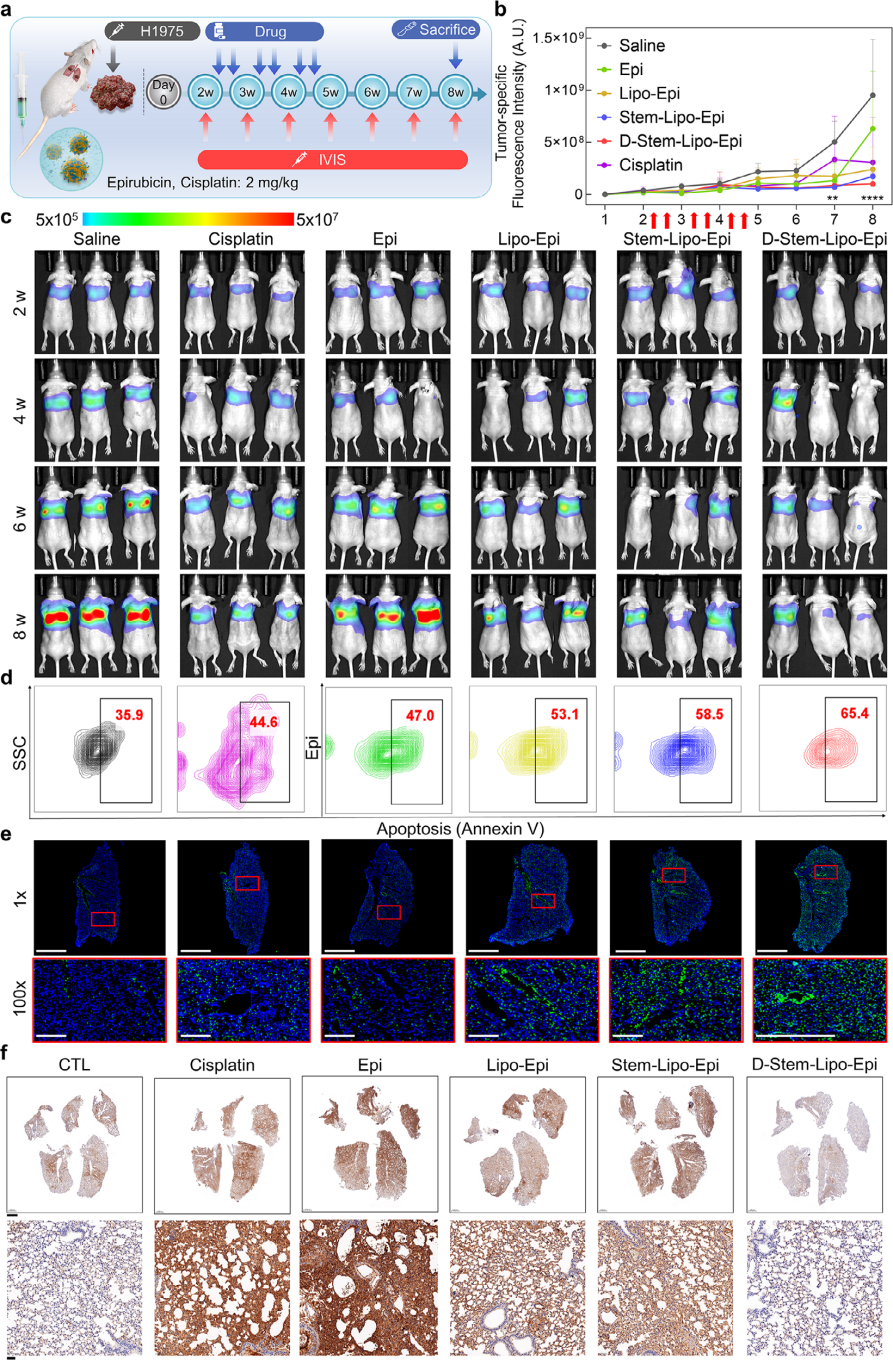
In vivo therapeutic effects in an orthotopic NSCLC mouse model. (a) Schematic illustration of the experimental schedule. (b) Quantification of the tumor burden using an in vivo imaging system (IVIS). Data are presented as mean ± SD (*n =* 5). ^**^ *p* < 0.01, ^****^ *p* < 0.0001 compared with Saline. (c) Representative serial images at 2, 4, 6, and 8 weeks. (d) Ex vivo flow cytometry showing epirubicin (Epi) fluorescence in dissociated tumor cells. (e) Representative images showing apoptotic cell staining in tumor regions. Scale bars, 2 mm (main) and 100 µm (inset). A.U., arbitrary units. (f) Representative images of Ki‐67 immunohistochemical staining in tumor sections. Scale bar, 1 mm (top) and 500 µm (bottom).

The in vivo therapeutic efficacy of the D‐Stem‐Lipo‐Epi platform was evaluated using an orthotopic H1975 NSCLC mouse model. Mice were intravenously administered 2 mg kg^−1^ epirubicin twice weekly for a total of six injections (Figure 4a). Treatment was initiated at an early stage to simulate the clinical management of minimal residual disease and prevent rapid tumor progression. During the 3‐week treatment period, all drug formulations successfully controlled tumor growth (Figure 4b). The basal signals from tumor‐free mice were utilized to subtract background noise during quantitative assessments (Figure S2). However, the initial tumor control was not sustained, as most groups exhibited significant tumor relapse after treatment cessation. Remarkably, three of five mice treated with D‐Stem‐Lipo‐Epi demonstrated a lasting effect, and the tumors did not regrow until the end of the experiment (Figure 4c). This sustained remission suggests that D‐Stem‐Lipo‐Epi effectively eradicated the tumor, thereby preventing its transition to a refractory state. To further quantify this potent in vivo effect, tumors were harvested and analyzed using flow cytometry. Consistent with in vitro results, the tumor cells isolated from the D‐Stem‐Lipo‐Epi group showed the highest percentage of apoptotic cells. The therapeutic efficacy of D‐Stem‐Lipo‐Epi was superior to that of the standard chemotherapeutic agent cisplatin (Figure 4d). This superiority over cisplatin is noteworthy given that cisplatin remains a first‐line clinical standard despite its high systemic risks [49]. Finally, a terminal deoxynucleotidyl transferase dUTP nick end labeling (TUNEL) assay and Ki‐67 immunohistochemical staining confirmed widespread cancer cell death signals and significantly suppressed cancer cell proliferation by D‐Stem‐Lipo‐Epi, further validating the successful tumor eradication (Figure 4e,f).

### 2.5 Systemic Toxicity and Biodistribution

**FIGURE 5:**
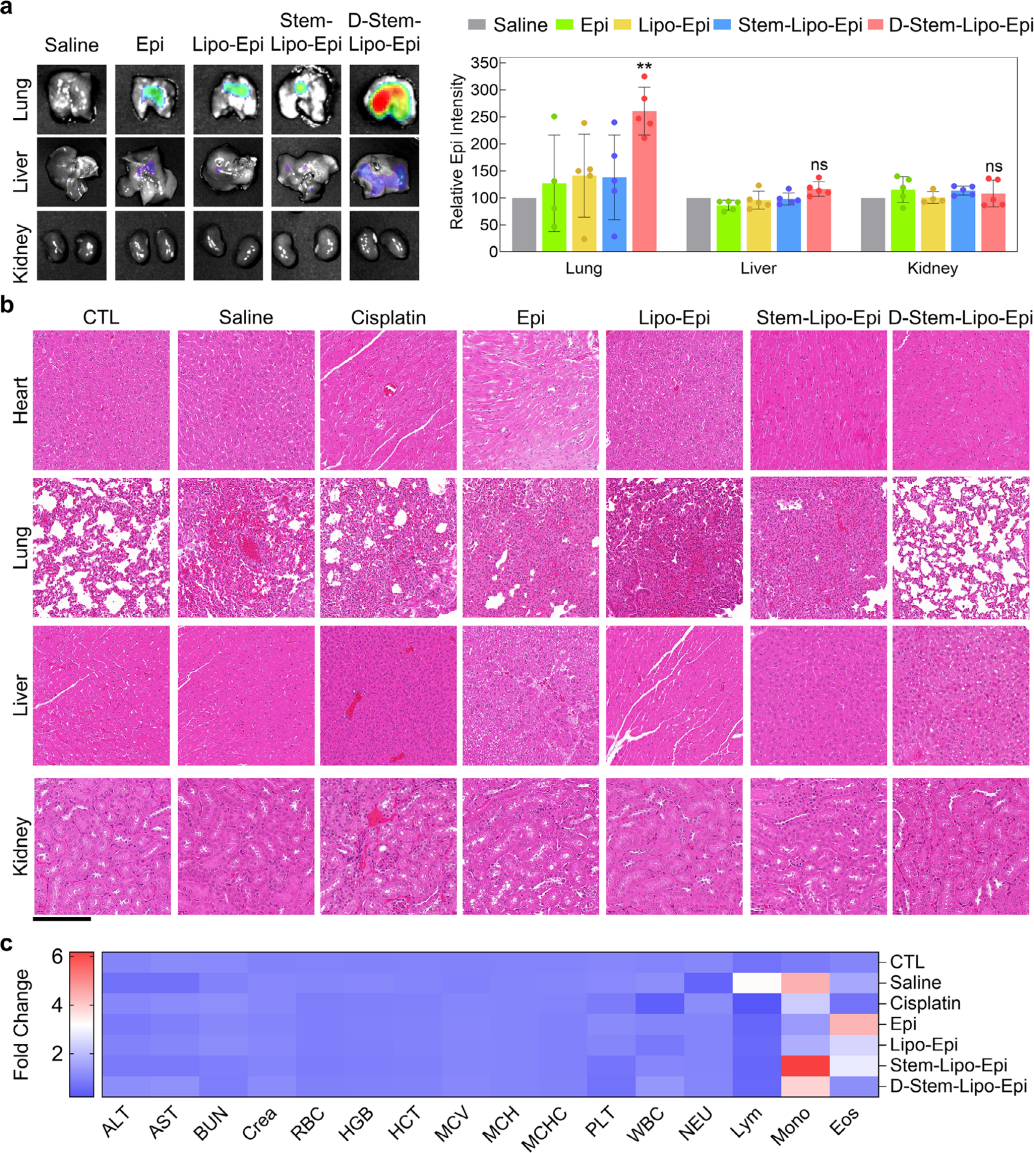
Biodistribution and systemic toxicity evaluation profile. (a) Ex vivo fluorescence images of the lung, liver, and kidney (left). The relative Epi intensity is quantified (right). Data are presented as mean ± SD (*n =* 5). ^**^ *p* < 0.01; ns, not significant. (b) Representative hematoxylin and eosin staining of major organs. Scale bar, 20 µm. (c) Heatmap at the terminal time point summarizing serum chemistry and complete blood count (*n =* 4), including alanine aminotransferase (ALT), aspartate aminotransferase (AST), blood urea nitrogen (BUN), creatinine (Crea), red blood cells (RBC), hemoglobin (HGB), hematocrit (HCT), mean corpuscular volume (MCV), mean corpuscular hemoglobin (MCH), mean corpuscular hemoglobin concentration (MCHC), platelets (PLT), white blood cells (WBC), neutrophils (NEU), lymphocytes (Lym), monocytes (Mono), and eosinophils (Eos). CTL, control; Epi, epirubicin.

To evaluate the organ accumulation and safety of nanoparticles in vivo, their biodistribution was analyzed. A distinct biodistribution profile was observed in the D‐Stem‐Lipo‐Epi group, which showed significantly higher accumulation in the lungs (Figure 5a). Quantitatively, this group demonstrated 1.8‐fold greater lung accumulation than the standard Lipo‐Epi group, whereas all other groups did not significantly differ. This enhanced lung‐specific targeting directly correlated with the superior tumor‐homing ability of D‐MSCs. In addition, immunohistochemistry analysis of major organs was performed (Figure 5b). In the lungs, most groups showed extensive tumor infiltration, whereas the tissue samples of the D‐Stem‐Lipo‐Epi group appeared clear of tumor cells and were equivalent to normal tissues. Regarding systemic toxicity, the livers from the cisplatin and epirubicin groups exhibited mild inflammation, whereas no significant histological abnormalities were observed in all other groups. Significant hepatotoxicity was observed in the cisplatin‐treated group, accompanied by a reduction in body weight that exceeded 20% by week 8 (Figure S2). The other major organs showed no signs of damage in any group. This was further supported by the blood biochemistry analysis (Figure 5c). The results revealed no significant changes in the liver function markers alanine aminotransferase (ALT) and aspartate aminotransferase (AST) or the kidney function markers blood urea nitrogen (BUN) and creatinine. Likewise, no significant changes were observed in other key parameters, including red blood cells, hemoglobin, hematocrit, mean corpuscular volume, mean corpuscular hemoglobin, mean corpuscular hemoglobin concentration, platelets, and white blood cells, including their differentials (neutrophils, monocytes, and eosinophils). Elevated lymphocyte counts in tumor‐bearing mice were restored to the normal range following treatment, likely due to a reduced tumor burden.

Taken together, these results demonstrate that the effective therapeutic dose used in this study (2 mg kg^−1^) is well within a safe range, with only minor off‐target effects [50]. The ability to achieve substantial anticancer effects at this low, nontoxic concentration underscores the advantage of the D‐Stem‐Lipo‐Epi approach by achieving a lung tumor‐targeted drug delivery system.

### 2.6 Transcriptomic Analysis of Dexa‐Primed MSCs

**FIGURE 6:**
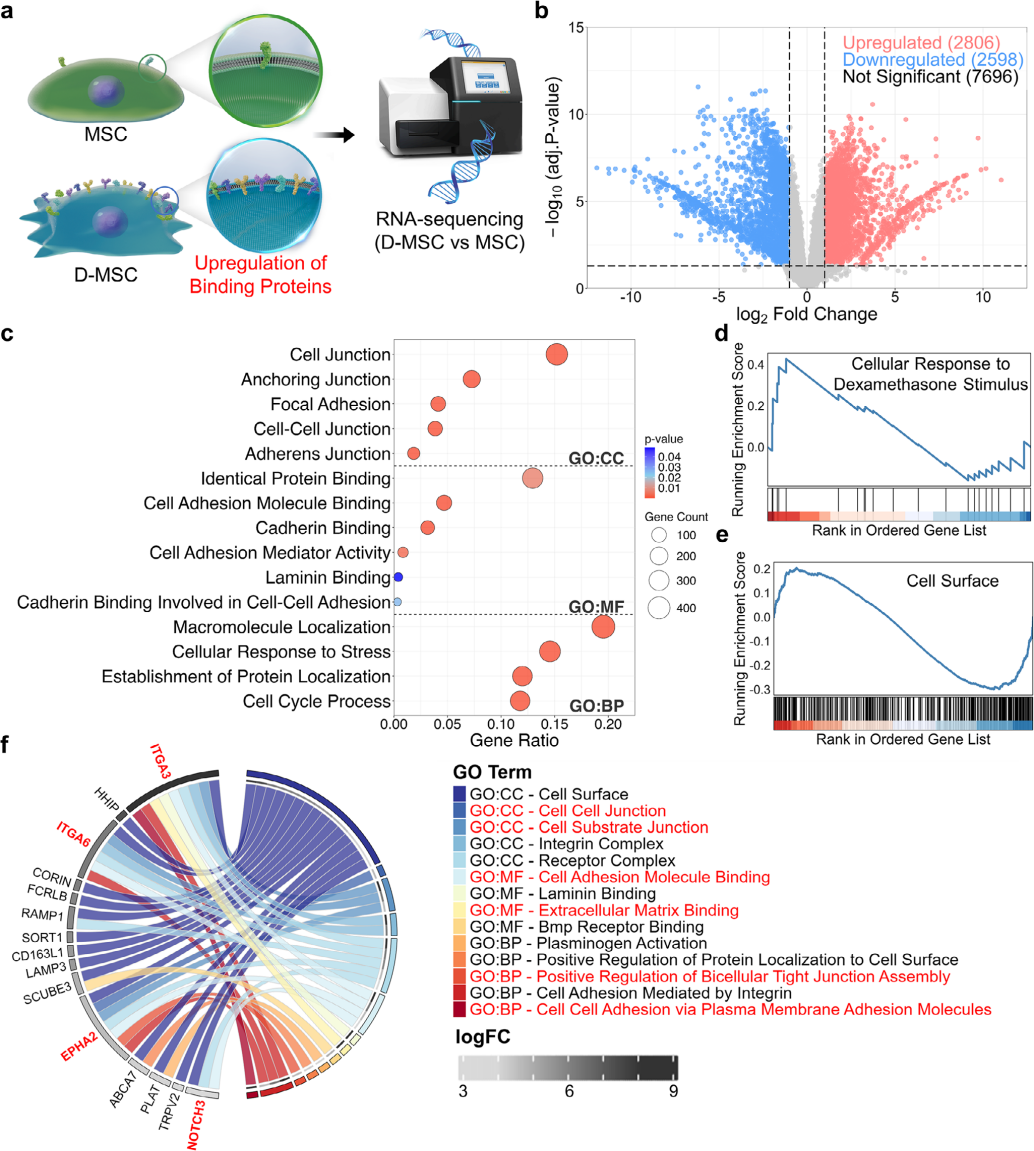
Transcriptomic analysis to elucidate upregulation of cell‐adhesion genes. (a) Schematic overview of the mRNA‐seq analysis comparing naïve MSCs and D‐MSCs. (b) Volcano plot illustrating differentially expressed genes (DEGs). Among a total of 13 100 analyzed genes, 2806 were upregulated (red), and 2598 were downregulated (blue), based on |logFC| > 1 and adjusted *p*‐value < 0.05. (c) Gene ontology (GO) enrichment analysis for upregulated DEGs, categorized by cellular component (CC), molecular function (MF), and biological process (BP). (d, e) Gene set enrichment analysis running enrichment score plot for the (d) “Cellular response to dexamethasone stimulus” and (e) “Cell surface” gene sets. (f) Chord plot illustrating the relationship between the top 15 upregulated cell surface genes and significantly associated GO terms. *p*‐value < 0.05. FC, fold change.

To elucidate the molecular mechanism underlying the enhanced tumor‐targeting ability of D‐MSCs, transcriptomic analysis was performed using mRNA sequencing (mRNA‐seq; Figure 6a and Figure S3). This approach is particularly relevant considering the vesicle production method. In conventional exosome biogenesis, the protein composition of the final vesicle can differ substantially from that of the plasma membrane of the parent cells [51, 52]. In contrast, the ectosome production method used in this study physically extrudes the cell membrane, which ensures that the surface protein profile is preserved on the resulting vesicles [38, 39]. This allows the identification of key factors responsible for the enhanced tumor‐targeting phenotype using mRNA‐seq results from parent cells.

The mRNA‐seq analysis confirmed that dexamethasone treatment induced a distinct transcriptomic shift, which was visually represented by a clear separation in the principal component analysis and the heatmap visualization (Figure S3). The analysis of differentially expressed genes revealed that 2806 genes were significantly upregulated, and 2598 genes were downregulated in D‐MSCs, as shown in the volcano plot (Figure 6b). Focusing on the upregulated genes, a gene ontology (GO) analysis revealed a significant enrichment of pathways related to cell–cell and cell–matrix interactions (Figure 6c). Gene set enrichment analysis (GSEA) was performed to further characterize transcriptomic changes. As expected, the analysis showed positive enrichment of the cellular response to the dexamethasone stimulus gene set (Figure 6d). The results also indicated an altered expression profile of cell surface proteins, suggesting a substantial rearrangement of the cell membrane in response to dexamethasone treatment (Figure 6e). To identify the most functionally relevant candidates among the surface proteins, the upregulated membrane proteins and their associated GO terms were further analyzed (Figure 6f). Chord plot analyses highlighted several key candidates, including EPHA2, NOTCH3, integrin subunit alpha 3 (ITGA3), and integrin subunit alpha 6 (ITGA6). These proteins are not only expressed on the cell surface but are also associated with crucial functions, such as cell adhesion, which might enhance the tumor‐targeting effect of D‐Stem‐Lipo‐Epi. Furthermore, upregulation of these proteins was confirmed at the translational level using western blot and flow cytometry analysis (Figure S4).

### 2.7 Identification of Key Mediators of Tumor Targeting

**FIGURE 7:**
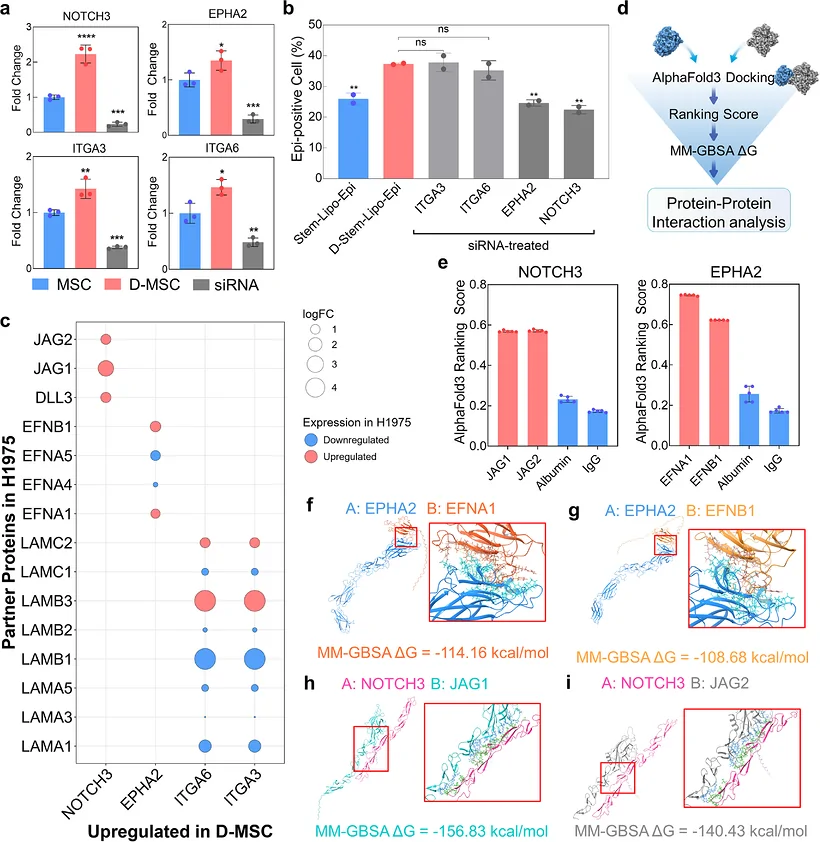
Functional and computational validation of key upregulated surface proteins. Quantitative real‐time polymerase chain reaction analysis of the indicated genes. Data are presented as mean ± SD (*n =* 3). ^*^ *p* < 0.05, ^**^ *p* < 0.01, ^***^ *p* < 0.001, ^****^ *p* < 0.0001. (b) Effect of siRNA knockdown in D‐MSCs on the cellular uptake (*n =* 3). (c) Dot plot of potential protein‐protein interactions between upregulated surface proteins on D‐MSCs against their potential binding partners on H1975 cells. (d) Schematic illustration of protein–protein docking and interaction analysis. (e) Model confidence calculated using AlphaFold 3 (*n =* 5). (f–h) Visualization and binding free energies (ΔG, kcal mol^−1^) for (f) EPHA2–ENFA1, (g) EPHA2–EFNB1, (h) NOTCH3–JAG1, and (i) NOTCH3–JAG2. FC, fold change; ns, not significant; EFNA1, ephrin‐A1; EFNB1, ephrin‐B1; JAG1, jagged‐1; JAG2, jagged‐2.

To investigate the functional necessity of the identified candidate proteins, targeted silencing of each gene was performed in MSCs using siRNAs prior to dexamethasone treatment. The successful knockdown of all four membrane proteins (EPHA2, NOTCH3, ITGA3, and ITGA6) was confirmed (Figure 7a). Stemsomes were then derived from each siRNA‐treated D‐MSC to generate the modified D‐Stem‐Lipo‐Epi. Subsequent cellular uptake assays yielded distinct results (Figure 7b). Knockdown of EPHA2 or NOTCH3 resulted in a significant reduction in cellular uptake, reverting it to the baseline level observed with non‐primed Stem‐Lipo‐Epi. In contrast, the suppression of ITGA3 or ITGA6 did not significantly diminish the uptake efficiency, which was equivalent to that of standard D‐Stem‐Lipo‐Epi.

To investigate potential PPIs, a bioinformatics analysis was performed using the Omnipath database (Figure S5) [53]. Potential interaction candidates were filtered based on their subcellular localization to the cell surface and extracellular matrix. These candidates were further refined through a literature‐based selection process to prioritize major interaction pairs and exclude false positives. The resulting dot plot visualized the fold change of proteins in H1975 cells in relation to the upregulated proteins identified in D‐MSCs (Figure 7c). While this analysis suggested that ITGA3 and ITGA6 are strong candidates for binding to multiple partner proteins in H1975 cells, the functional assay demonstrated converse results. In contrast, the suppression of EPHA2 and NOTCH3 substantially reduced uptake to baseline levels, identifying these proteins as the primary mediators of the tumor‐targeting effect. However, the extent of this effect suggests that their contribution may not be limited to simple physical adhesion. Since EPHA2 and NOTCH3 are critical signaling molecules in MSCs, their knockdown may have altered other cellular activities that indirectly influenced the overall adhesion process beyond the direct loss of the two binding sites [54, 55].

To investigate contributions beyond protein expression, a computational simulation was performed (Figure 7d). The protein structures of EPHA2 and NOTCH3 and their respective ligand families, including ephrin‐A1 (EFNA1), ephrin‐B1 (EFNB1), Jagged‐1 (JAG1), and Jagged‐2 (JAG2), were selected. The AlphaFold 3 database and Schrödinger suite were used to prepare the protein structures and perform protein–protein docking to predict binding poses [56, 57]. To further validate the structural reliability of these interactions, the AlphaFold 3 confidence score was calculated as a weighted combination of the interface‐predicted template modeling score (ipTM) and the predicted template modeling score (pTM). The main candidates achieved high confidence scores exceeding 0.55, whereas nonspecific controls, such as immunoglobulin G and albumin, yielded significantly lower scores ranging from 0.15 to 0.25 (Figure 7e). Generated interaction maps visualized the binding interfaces, which appeared to be mainly driven by hydrogen bonds and pi stacking (Figure S6). Finally, binding free energies were assessed by calculating the Gibbs free energy (ΔG) via the molecular mechanics generalized born surface area (MM‐GBSA) method. The simulation results demonstrated that EPHA2 showed favorable ΔG values with EFNA1 (−114.16 kcal mol^−1^) and EFNB1 (−108.68 kcal mol^−1^), whereas NOTCH3 exhibited even more potent interactions with JAG1 (−156.83 kcal mol^−1^) and JAG2 (−140.43 kcal mol^−1^; Figure 7f–i). While these MM‐GBSA‐derived absolute values often overestimate experimental affinities due to the solvent‐exposed nature of protein interfaces, they fall within a range indicative of a strong interaction potential when compared to various PPI benchmarks [58]. The binding potential is particularly important, given that they show a substantially higher fold change in H1975 NSCLC cells than in non‐malignant BEAS‐2B cells. These results collectively suggest that upregulated EPHA2 and NOTCH3 on the D‐MSC surface preferentially mediate specific interactions with their tumor‐associated partners, rather than nonspecific binding in the plasma.

## 3 Discussion

In this study, a highly effective drug for lung cancer was developed by fusing drug‐loaded liposomes with nanovesicles derived from dexamethasone‐treated MSCs. This novel platform induced effective and durable tumor regression in vivo, which was attributed to its enhanced lung‐targeting efficiency and safety profile. The novelty of this work lies in its unique application of dexamethasone. To the best of our knowledge, this is the first study to repurpose dexamethasone, not for its conventional anti‐inflammatory or osteogenic functions, but as a tool to engineer the surface of cell‐derived nanovesicles. Through transcriptomic analysis and in vitro validation, several key mediators were identified, including cell adhesion molecules, such as ITGA3, ITGA6, EPHA2, and NOTCH3. However, functional verification through siRNA‐mediated knockdown demonstrated that their individual contributions to the tumor‐targeting process were distinct. The absence of ITGA3 and ITGA6 knockdown effects could be attributed to several factors. First, unlike EPHA2 and NOTCH3, which bind directly to ligands on the cancer cell surface, integrins primarily mediate adhesion to extracellular matrix components, such as laminin. This interaction might be less critical during the initial cell internalization step. Second, the well‐documented functional redundancy within the integrin family may have allowed other integrin subunits to compensate for the loss of ITGA3 and ITGA6 [59].

Although the present computational analysis focused primarily on two proteins owing to limitations in protein structure preparation and computational resource requirements, the extensive bioinformatics data imply a much broader multivalent PPI network. This suggests that the interactions between the D‐Stemsome surface and cancer cells likely involve concurrent binding events beyond a single receptor–ligand pair. These findings highlight that the targeting efficiency is not dictated by a few known ligands but by a complex interplay of partner expression, binding affinity, and the biological context of the interaction. However, the limitations of this computational approach should be acknowledged and interpreted carefully. While these physics‐based suites are well established for predicting small‐molecule binding, their predictive accuracy for complex PPIs is considered less robust [60]. Nevertheless, the primary value of this simulation lies not in its absolute predictive accuracy but in its comparative analysis of multiple ligands against individual receptors, such as EPHA2 and NOTCH3. This approach revealed that partner proteins in H1975 were not only overexpressed but also exhibited strong binding energies.

The mechanistic advantage of the multivalent PPI network is further supported by established principles of binding avidity in nanoparticle‐based delivery systems [61, 62]. Previous studies have demonstrated that multivalent displays can enhance binding avidity by several orders of magnitude, up to 170 000‐fold compared to monovalent counterparts, which is primarily driven by an exponential decrease in the dissociation rate rather than an increased rate of endocytosis. This suggests that the upregulated surface proteins on H1975 cells, such as EPHA2 and NOTCH3, collectively increase the residence time of D‐Stemsomes on the cancer cell surface by providing multiple simultaneous binding points that prevent rapid dissociation. Furthermore, the efficiency of such multivalent interactions often depends on a matching pattern between ligand and receptor densities, where a minimum of ∼1.6 bonds and a specific receptor‐to‐ligand density ratio are required for receptor‐mediated endocytosis. Our findings likely reflect this threshold effect, wherein the Dexa‐induced surfaceome reprogramming ensures that the D‐Stemsome surface exceeds the requisite binding threshold for NSCLC cells while potentially minimizing off‐target binding to normal cells that exhibit only baseline receptor expression.

The profound advantage of the dexamethasone‐priming strategy is its ability to amplify the entire multifaceted biological system, which offers clear benefits over traditional methods that rely on a single ligand. By utilizing a widely available compound, such as dexamethasone, to augment targeting, this approach represents a simple and highly translational strategy for nanomedicines targeting lung cancer. These findings present a new strategy for the development of MSC‐based therapies with high clinical applicability.

## 4 Conclusion

In summary, the dexamethasone treatment of MSCs provides a simple yet highly translational approach for nanomedicines by amplifying a biological protein interaction system. This multivalent surface engineering overcomes existing targeting limitations, establishing a novel strategy for the development of MSC‐based targeted therapies with high clinical applicability.

## 5 Experimental Section

### 5.1 Cell Culture

Human adipose‐derived MSCs (CEFO‐ADMSC) were purchased from CEFO Co., Ltd. (Seoul, South Korea) and cultured in CoreMAX XF series media (CM001‐00, CORECELL, Seoul, South Korea) supplemented with the provided proprietary additives according to the manufacturer's protocol. The human NSCLC cell line H1975 (CRL‐5908) was obtained from the American Type Culture Collection (RRID: CVCL_1511, ATCC, Manassas, VA, USA) and maintained in Dulbecco's modified Eagle's medium (DMEM) with high glucose (11995‐065, Gibco, Grand Island, NY, USA), supplemented with 10% FBS (16000‐044, Gibco) and 1% antibiotic solution (100 units mL^−1^ penicillin and 100 µg mL^−1^ streptomycin; 10378016, Gibco). All cell lines were incubated at 37°C in a humidified atmosphere containing 5% CO_2_.

### 5.2 Dexamethasone Treatment

Human adipose‐derived MSCs were seeded at a density of 1 × 10^6^ cells per 100 mm culture dish and cultured overnight to allow for cell adhesion and stabilization. Then, MSCs were incubated with complete culture medium supplemented with 200 nм dexamethasone (Sigma–Aldrich, St. Louis, MO, USA) for 24 h at 37°C under a humidified atmosphere containing 5% CO_2_. The resulting D‐MSCs were collected and used for stemsome isolation or further experimental analyses.

### 5.3 Migration Analysis

Migration of MSCs was assessed as previously described [39]. Briefly, H1975 cells were seeded at a density of 5 × 10^4^ cells per well in 24‐well plates (CLS3527, Corning, Corning, NY, USA) and cultured overnight in 500 µL complete medium. Subsequently, MSCs and D‐MSCs were seeded separately at a density of 5 × 10^4^ cells in the upper chambers of 24‐well transwell inserts (polycarbonate membrane, 8 µm pore size; #3422, Corning) containing 200 µL of complete medium. After incubation for 3 h, nonmigrated cells remaining on the upper side of the membrane were gently removed. Migrated cells adhering to the lower surface of the transwell membrane were fixed with ice‐cold 100% methanol for 1 min at 4°C. After three washes with phosphate‐buffered saline (PBS), the cells were stained with 4,6‐diamidino‐2‐phenylindole (DAPI; Invitrogen, Carlsbad, CA, USA) for 30 min at room temperature. Images of migrated cells were captured using an EVOS M7000 microscope (Thermo Fisher Scientific, Waltham, MA, USA) at 1000 × magnification. Ten randomly selected high‐power fields per membrane were analyzed, and the average number of migrated MSCs was calculated and expressed as cells per high‐power field to evaluate their chemotactic migration toward H1975 cells.

### 5.4 SDS‐PAGE

Proteins were extracted from MSCs, D‐MSCs, stemsomes, D‐Stemsome, Stem‐Lipo, and D‐Stem‐Lipo using RIPA buffer (GenDEPOT, Barker, TX, USA) according to the manufacturer's instructions. Bradford protein assays were performed using Pierce Bradford Plus Protein Assay Reagent (Thermo Fisher Scientific) according to the manufacturer's instructions. Based on the protein concentration, the sample amount used for SDS‐PAGE was determined. SDS‐PAGE samples were prepared by PAGESTA Reducing 5X SDS‐PAGE Sample Buffer (GeneAll Biotechnology, Seoul, South Korea). Electrophoresis was performed at 100 V for 90 min, and the gels were stained using Pierce Bradford Plus Protein Assay Reagent (Thermo Fisher Scientific) with gentle orbital shaking overnight. The gels were then destained in destain solution (40% methanol and 10% acetic acid), and images of the gels were captured using an Amersham ImageQuant 800 (Cytiva, Marlborough, MA, USA).

### 5.5 Generation of H1975_Luci–GFP_ Cells

H1975 cells were seeded at a density of 3 × 10^5^ cells per well in 6‐well plates and cultured overnight. Subsequently, a mixture of 1 mL lentiviral supernatant (#GlowCell‐16p‐1, Biosettia, San Diego, CA, USA) encoding luciferase (Luci) and green fluorescent protein (GFP; 1 × 10^5^ viral particles per well), combined with 2 mL of complete culture medium, was added. After incubation for 24 h, the medium was replaced with a fresh medium, and the cells were incubated for 72 h. Transduced cells were selected by puromycin (1 µg mL ^−1^; Sigma–Aldrich). Transduced H1975_Luci–GFP_ cells were continuously cultured in medium containing puromycin (0.3 µg mL^−1^) for one month to ensure stable selection. The successful establishment of H1975 _Luci–GFP_ cells was confirmed by bioluminescence imaging using an IVIS imaging system (PerkinElmer, Waltham, MA, USA) following the administration of D‐luciferin (150 mg mL^−1^; Gold Biotechnology, St. Louis, MO, USA).

### 5.6 Orthotopic Lung Cancer Model

Female BALB/c nude‐Foxn‐1‐nu mice were obtained from Orient Bio (Seoul, South Korea). Upon arrival, five mice per cage were housed in a laminar airflow facility maintained at a controlled temperature (22 ± 2°C), relative humidity (55% ± 5%), and a 12‐h light/dark cycle (lights on at 7:00 a.m.). All animals were acclimatized to pathogen‐free conditions for 7–14 days prior to the experiment. For orthotopic modeling, H1975_Luci–GFP_ cells were prepared in PBS at a concentration of 1 × 10^6^ cells per 200 µL. The mice were anesthetized by isoflurane inhalation, and the prepared H1975_Luci–GFP_ cell suspension was intravenously injected via the tail vein. Immediately following the injection, the animals were returned to clean cages and maintained under pathogen‐free conditions for subsequent experimental analyses.

### 5.7 Synthesis of Lipid Nanoparticles

Liposomes were synthesized using a mixture of 1,2‐dioleoyl‐sn‐glycero‐3‐phosphocholine, cholesterol, and 1,2‐distearoyl‐sn‐glycero‐3‐phosphoethanolamine‐N‐[methoxy(polyethylene glycol)‐2000] (Avanti Polar Lipids, Alabaster, AL, USA) in a molar ratio of 60:35:5. Lipids were initially dissolved in 99.9% ethanol, followed by sonication for 10 min at 4°C. The ethanol–lipid solution and aqueous phase were then mixed using a microfluidic‐based lipidnanoparticle synthesis system (NEO NANOTECH, Seoul, South Korea), maintaining a flow rate ratio of the aqueous to organic phases at 1:10 and a total flow rate of 8 mL min^−1^. The resultant liposomes were diluted 1:10 with PBS and centrifuged at 3000 rpm for 20 min at 4°C using Amicon Ultra centrifugal filters (Millipore, Billerica, MA, USA) with a molecular weight cutoff of 100 kDa.

### 5.8 Encapsulation of Epirubicin in Lipid Nanoparticles

Epirubicin encapsulation in liposomes was performed using an ammonium sulfate gradient method as previously described [40,63]. Briefly, liposomes were prepared by incorporating 250 mм ammonium sulfate solution into the aqueous phase. Free ammonium sulfate was removed using centrifugal filters. Subsequently, epirubicin solution (1 mg mL^−1^) was mixed and incubated at 50°C for 30 min. The resulting Lipo‐Epi was further purified by centrifugation (3000 rpm, 20 min, 4°C) using molecular weight cutoff 100 kDa filters. The purified Lipo‐Epi nanoparticles were stored at 4°C until further use.

### 5.9 Stem Cell Membrane Extraction

Stemsomes were isolated following a previously established cytochalasin B‐based method [38]. Briefly, MSCs and D‐MSCs were washed twice with PBS and then incubated for 25 min in serum‐free DMEM supplemented with 10 µg mL^−1^ cytochalasin B (Sigma–Aldrich). Subsequently, the cells were detached and vortexed for 1–5 min to facilitate the formation of membrane vesicles. The resulting cell suspension was centrifuged at 1000 rpm for 5 min to remove intact cells and debris. The supernatants were further centrifuged at 4000 rpm for 15 min to yield purified stemsomes from the pellet. The collected stemsomes were resuspended in PBS and stored at 4°C for subsequent experiments.

### 5.10 Preparation of Stemsome–Liposome Nanoparticles

To prepare nanoparticles, Lipo‐Epi was mixed with stemsomes or D‐Stemsome (derived from either MSCs or D‐MSCs) and fused by extrusion using an Avanti Mini‐Extruder (Avanti Polar Lipids). The suspension was passed 10 times through a 1.0 µm polycarbonate membrane to generate the final fusion nanoparticles (Stem‐Lipo‐Epi and D‐Stem‐Lipo‐Epi, respectively).

### 5.11 Characterization of the Generated Nanoparticles

The encapsulation of epirubicin within Lipo‐Epi and Stem‐Lipo was quantified by measuring absorbance at 480 nm using a UV–vis spectrophotometer (Libra S50, Biochrom, Cambridge, UK). The particle size distribution and zeta potential of the various nanoparticles were determined using a dynamic light scattering analyzer (Litesizer 500, Anton Paar, Graz, Austria).

### 5.12 Energy‐Dispersive X‐Ray Spectroscopy Mapping

Stem‐Lipo fusion was evaluated by STEM–EDS mapping as previously described [42]. Briefly, to specifically label the stem cell membrane, samples were incubated with an anti‐CD90–streptavidin conjugate, followed by incubation with 5 nm gold nanoparticles (#741949, Merck, Darmstadt, Germany) functionalized with biotin. After removing unbound gold nanoparticles by centrifugation, the samples were negatively stained with uranyl acetate, loaded onto copper grids, and air‐dried. The morphology and elemental distribution were examined by STEM–EDS (ARM200F, JEOL, Tokyo, Japan).

### 5.13 Fluorescence Resonance Energy Transfer Assay

Stem‐Lipo fusion was further evaluated using a FRET assay. For FRET analysis, stemsomes were labeled using DiI (Thermo Fisher Scientific) as the fluorescent donor (excitation/emission: 549/565 nm), and liposomes were labeled with DiD (Thermo Fisher Scientific) as the fluorescent acceptor (excitation/emission: 644/665 nm), following the manufacturer's instructions. Upon fusion, the fluorescence energy transfer between DiI‐labeled stemsomes and DiD‐labeled liposomes was detected by measuring the fluorescence intensity at excitation and emission wavelengths of 549 and 665 nm, respectively, using an in vivo imaging system (IVIS Lumina III; PerkinElmer).

### 5.14 Analysis of Cellular Uptake

H1975 cells were seeded at a density of 1 × 10^5^ cells per well. After overnight incubation, cells were pretreated with the endocytic inhibitors chlorpromazine, genistein, or EIPA (25, 200, and 20 µм, respectively) for 1 h. Then, cells were treated with nanoparticles for another 4 h. After incubation, the cells were detached with 1X trypsin‐EDTA buffer, centrifuged at 1,500 rpm for 5 min, and resuspended in staining buffer. Cellular uptake was evaluated using flow cytometry (BD LSRFortessa, BD Biosciences), and the data were analyzed using FlowJo software (FlowJo, Ashland, OR, USA).

### 5.15 Endosomal Escape

The intracellular trafficking of epirubicin‐loaded formulations was evaluated by immunofluorescence staining of early and late endosomes. The cells were treated with free epirubicin, Lipo‐Epi, Stem‐Lipo‐Epi, or D‐Stem‐Lipo‐Epi for 2, 12, or 24 h, followed by fixation with 2% paraformaldehyde. Early and late endosomes were stained with anti‐EEA1 (ab2900, Abcam, Cambridge, UK) and anti‐M6PR (ab2733, Abcam) antibodies, respectively, followed by Alexa Fluor‐conjugated secondary antibodies (ab150075 and ab150108, Abcam). Nuclei were counterstained with DAPI. Fluorescent images were acquired using an EVOS M7000 imaging system (Thermo Fisher Scientific).

### 5.16 Cell Viability Assay

Cell viability was evaluated using a Cell Counting Kit‐8 (CCK‐8; Abcam) assay. Briefly, cells were seeded in 96‐well plates at a density of 5 × 10^3^ cells per well and incubated overnight at 37°C. For therapeutic efficacy evaluation, H1975 cells were treated with nanoparticles (equivalent to 0.5 µg mL^−1^ epirubicin) for 72 h. For the evaluation of dexamethasone toxicity, MSCs were treated with varying concentrations of dexamethasone (0–1600 nм) for 24 h. Following the respective treatments, 10 µL of CCK‐8 solution was added to each well, the cells were incubated for 2 h at 37°C, and absorbance was measured at 460 nm (UVM 340, Biochrom). Cell viability (%) was calculated relative to that of control wells.

### 5.17 Apoptosis Analysis by Annexin V Assay

Apoptosis was assessed using Annexin V. H1975 cells were seeded at 3 × 10^5^ cells per well in six‐well plates and incubated overnight. The cells were then treated with nanoparticles (equivalent to 0.5 µg mL^−1^ epirubicin) for 48 h. After treatment, the cells were harvested and washed with PBS. The cells were subsequently stained with Annexin V‐FITC for 10 min at room temperature. Following staining, the cells were washed with binding buffer, resuspended in staining buffer, and immediately analyzed by flow cytometry (BD LSRFortessa). Apoptotic cell populations were quantified using the FlowJo software.

### 5.18 In Vitro Drug Stability

The stability of Stem‐Lipo‐Epi was simultaneously monitored under PBS (pH 7.4), PBS (pH 6.5), acetate buffer saline (pH 5.0), and 10% FBS conditions by measuring the hydrodynamic size of each aliquot at specified time intervals (0, 2, 4, 6, 12, and 24 h) using a dynamic light scattering analyzer (Litesizer 500, Anton Paar, Graz, Austria).

### 5.19 Bioluminescence Imaging

In vivo bioluminescence imaging was performed using an IVIS Lumina III imaging system (PerkinElmer) to monitor luciferase‐expressing H1975_Luci–GFP_ tumor progression. Imaging was performed every week throughout the experimental period. The mice were anesthetized with 2.5% isoflurane in air, and D‐luciferin (150 mg kg^−1^) was intravenously administered via the tail vein immediately before imaging. Animals were placed in a light‐tight imaging chamber, and luminescence signals were acquired over a 10 s exposure time. The images were analyzed using Aura Imaging software (Spectral Instruments Imaging, Tucson, AZ, USA). Bioluminescence intensity was quantified as photon flux (photons s^−1^ cm^−2^ sr^−1^), which was calculated from the regions of interest placed over the thoracic cavity.

### 5.20 In Vivo Antitumor Efficacy

To evaluate the therapeutic efficacy of the formulations, H1975_Luci–GFP_‐bearing mice with established tumors (defined as a bioluminescence intensity >1 × 10^7^ photons s^−1^) were randomly assigned into six groups. The mice received intravenous injections twice weekly for three consecutive weeks. The treatment groups were as follows: (i) saline, (ii) cisplatin (2 mg kg^−1^), (iii) free epirubicin, (iv) Lipo‐Epi, (v) Stem‐Lipo‐Epi, and (vi) D‐Stem‐Lipo‐Epi (all at 2 mg kg^−1^ epirubicin equivalent).

### 5.21 Histological and Immunohistochemical Analysis

After completion of the final bioluminescence imaging, all mice in the experimental groups were euthanized, and tissues from the tumor and major organs, including the heart, liver, spleen, lung, and kidneys, were harvested for ex vivo imaging and histological evaluation. Tissues were fixed in 10% neutral‐buffered formalin, embedded in paraffin, and sectioned into 4 µm‐thick slices. Paraffin‐embedded tissues were deparaffinized, rehydrated, and stained with hematoxylin and eosin following standard protocols. The TUNEL assay was performed using a commercial kit (G3250; Promega, Madison, WI, USA). After deparaffinization, rehydration, and proteinase K treatment, the endogenous peroxidase activity was blocked. The sections were equilibrated and incubated with TdT enzyme according to the manufacturer's instructions. After washing, the sections were mounted using a DAPI‐containing mounting medium (ab104239, Abcam). For immunohistochemistry, formalin‐fixed paraffin‐embedded sections were deparaffinized and processed using the Leica BOND RXm automated immunostainer (Leica Microsystems, Wetzlar, Germany). Heat‐induced epitope retrieval (EDTA buffer, pH 9.0), endogenous peroxidase blocking, and non‐specific binding prevention were performed according to the manufacturer's instructions. The primary antibody against Ki67 (ab16667, Abcam) was applied at a 1:500 dilution. Detection was performed using the Bond Polymer Refine Detection system (DS9800, Leica Microsystems).

### 5.22 Toxicological Assessment

To assess systemic toxicity, blood samples were collected from the orbital sinus of each mouse in heparinized collection tubes (5 units mL^−1^ heparin). Samples were maintained on ice, and a complete blood count was performed using an automated blood analyzer (Sysmex F‐820, Toa Medical Electronics, Japan). For serum chemistry, blood was allowed to coagulate at room temperature and centrifuged at 2000 × *g* for 15 min at 4°C. The resulting serum was transferred to fresh tubes, and the levels of ALT, AST, BUN, and creatinine were measured using standard clinical chemistry reagent kits following the manufacturer's protocols.

### 5.23 Gene Knockdown Using siRNA

Small interfering RNAs (siRNAs) specifically targeting human *ITGA3*, *ITGA6*, *EPHA2*, and *NOTCH3* were synthesized and purchased from Bioneer (Daejeon, South Korea). The sequences for siRNAs are summarized in Table S1. Transfection was performed 4 h prior to dexamethasone treatment using Lipofectamine 3000 (Invitrogen, Carlsbad, CA, USA) following the manufacturer's recommended protocol. The efficacy of the gene knockdown was confirmed by quantitative real‐time PCR (qPCR) before proceeding with stemsome isolation.

### 5.24 Reverse Transcription Quantitative PCR

RNA was isolated using the RNEasy Mini Kit (QIAGEN, Hilden, Germany) following the manufacturer's protocol. The RNA quality, total RNA yield, and 260/280 and 260/230 ratios were measured using a NanoDrop spectrophotometer (Thermo Fisher Scientific, MA, USA). Total RNA was reverse‐transcribed into complementary DNA using a PrimeScript first Strand cDNA Synthesis Kit following the manufacturer's protocol (Takara, Shiga, Japan). The complementary DNA template was quantified by real‐time PCR using SYBR Green fluorescence (Takara) on a CFX384 (Bio‐Rad, Hercules, CA, USA). The primers used are listed in Table S2.

### 5.25 Protein Expression Analysis

MSCs and D‐MSCs (2 × 10^5^ cells per well) were seeded into six‐well plates and incubated at 37°C to allow cell adhesion. After the indicated treatment period, proteins were isolated using lysis buffer containing 20 mm Tris, 150 mm NaCl, 2 mm EDTA, 1% Triton X‐100, and a protease inhibitor mixture. Protein concentration was quantified using the Bradford assay (5000207, Quick Start BSA Standard, Bio‐Rad). Proteins were denatured in SDS sample buffer at 37°C for 30 min, and denatured protein samples (30 µg each) were subjected to SDS‐PAGE. Proteins were detected using antibodies against EPHA2 (MA547953, Invitrogen), NOTCH3 (701975, Invitrogen), and β‐actin (A3854, Sigma). Immunoreactive bands were visualized using an enhanced chemiluminescence solution (Thermo Fisher Scientific), and images were acquired using an Amersham ImageQuant 800 imaging system (29399481, Cytiva). For flow cytometry, cells were incubated with primary antibodies against ITGA3 (HPA008572, Sigma–Aldrich) and ITGA6 (sc‐374057, Santa Cruz Biotechnology) for 1 h at 4°C, followed by centrifugation to remove unbound antibody. Cells were then incubated with the appropriate secondary antibody for 30 min at 4°C and centrifuged again. Fluorescence was measured using a spectral cell analyzer (SA3800, Sony), and data were analyzed using FlowJo software (FlowJo, Ashland, OR, USA).

### 5.26 mRNA‐Seq

Total mRNA was extracted using the TRIzol reagent (Invitrogen) according to the manufacturer's instructions. RNA integrity and quality were verified using an Agilent 2100 Bioanalyzer (Agilent Technologies, Amstelveen, The Netherlands), and RNA concentration was quantified using an ND‐2000 spectrophotometer (Thermo Fisher Scientific). RNA libraries were prepared using the NEBNext Ultra II Directional RNA‐Seq Kit (New England BioLabs, Hitchin, UK). Poly(A) RNA selection was performed using a Poly(A) RNA Selection Kit (Lexogen, Vienna, Austria). Subsequently, purified mRNA samples were subjected to cDNA synthesis and fragmentation according to the manufacturer's protocol. Library indexing was performed using Illumina indices 1–12, followed by enrichment by PCR amplification. Library quality was assessed using a TapeStation HS D1000 Screen Tape (Agilent Technologies), and accurate library quantification was performed using a Library Quantification Kit on a StepOne Real‐Time PCR System (Life Technologies, Carlsbad, CA, USA). High‐throughput paired‐end sequencing (2 × 100 bp) was performed using the Illumina NovaSeq 6000 platform (Illumina, San Diego, CA, USA).

### 5.27 Bioinformatics Analysis

Raw sequencing data quality was evaluated using the FastQC software. Low‐quality reads (quality score < Q20) and adapter sequences were removed using the FASTXTrimmer and BBMap software. Cleaned reads were mapped to the human reference genome using TopHat software. Subsequent analyses were conducted using the R software (version 4.2, R Development Core Team). The data were processed using the edgeR package, low‐expression genes were filtered, and counts were normalized using the Trimmed Mean of M‐values (TMM) method. Differential gene expression analysis was performed using the limma‐voom pipeline, and functional enrichment analyses (GSEA and over‐representation analysis) were conducted using the clusterProfiler, fgsea, and gprofiler2 packages.

### 5.28 Protein–Protein Docking

Protein–protein docking of each surface protein was conducted using the AlphaFold server and Schrödinger suite (version 2025‐4; Schrödinger, New York, NY, USA). The prediction confidence and interface quality were evaluated by analyzing the predicted aligned error (PAE) matrix using the PAE viewer [64]. The predicted complex structures were subsequently imported into Schrödinger 2025‐3 for refinement. Prior to the energy calculations, all structures were preprocessed using the protein preparation wizard. This process included the addition of hydrogen atoms, removal of non‐protein atoms, and optimization of the hydrogen‐bonding network. Finally, restrained minimization was performed using the OPLS4 force field to relieve steric clashes while maintaining overall structural integrity. The stereochemical quality of the refined models was validated using Ramachandran plot analysis to confirm that the backbone torsion angles were within favorable regions.

### 5.29 MM‐GBSA Binding Free‐Energy Calculations

Binding free energy for each minimized protein complex was estimated using the Prime MM‐GBSA module. The VSGB implicit solvent model and the OPLS4 force field were applied to compute binding free energies (ΔG_bind_) under energy‐minimized conditions. Protein flexibility was disabled by setting the flexible‐residue distance cutoff to 0.0 Å, treating both receptor and ligand as rigid during energy minimization. Calculations were performed using the minimize sampling method, without applying additional constraints to the flexible residues. The total binding free energy was determined using the following standard Equation (1):

(1) $$\Delta \;G_{bind}=G_{complex}\;-\;\left(G_{receptor}+G_{ligand}\right)$$

where G_complex_, G_receptor_, and G_ligand_ represent the minimized potential energies of the optimized complex, receptor, and ligand, respectively. The representative docking poses were independently evaluated, and the mean ΔG value was used for comparative analysis among complexes.

### 5.30 Ethics

All animal experiments were performed with the approval of the Institutional Animal Care and Use Committee of Gachon University (Approval No. LCDI‐2024‐0051). The studies were conducted in strict accordance with the national guidelines for the care and use of laboratory animals and reported following the Animal Research: Reporting of In Vivo Experiments guidelines. Animal body weight and health status were monitored daily. The humane endpoint was defined as a weight loss of ≥ 20% relative to the pre‐injection baseline of the control group.

### 5.31 Statistical Analysis

Quantification was performed on data obtained from at least three independent biological experiments, and data are presented as mean ± standard deviation of the mean. To evaluate statistical significance, *P*‐values were calculated using one‐way analysis of variance followed by Tukey's post hoc test for multiple comparisons. Statistical significance was defined as *p* < 0.05, and exact *P* ‐values are provided in the respective figure legends. All statistical computations and raw data processing were performed using GraphPad Prism software (version 9.0; GraphPad Software, Boston, MA, USA) and R software (version 4.4.2; R Foundation for Statistical Computing, Vienna, Austria).

### 5.32 Declaration of AI and Software Use

The authors declare that no generative artificial intelligence was utilized at any stage of manuscript preparation, including for data visualization, illustrations, or image generation. For data, GraphPad Prism and R, utilizing the ggplot2 package, were employed. Molecular modeling and related visualizations were conducted using the Schrödinger Suite. All schematic illustrations were designed by professional illustrators at Bioartlab (Anyang, South Korea), and the final manuscript underwent comprehensive academic language editing by certified experts at Editage (Seoul, South Korea).

## Author Contributions

G.K., Y.L., and D.K. conceptualized the research. G.K. and Y.L. co‐wrote the original draft. D.K. supervised the drafting of the manuscript. G.K., I.A.K., Y.L., and D.K. visualized data. G.K., I.A.K., and B.S.J. synthesized and characterized nanoparticles. G.K. conducted cell experiments. G.K., I.A.K., and S.‐R.L. designed and conducted the animal study. J.‐H.P. analyzed toxicity data, and H.S.K performed RT‐qPCRs. Y.L. performed transcriptomic and flow cytometry analyses. I.A.K. and Y.L. led the mechanism‐of‐action investigation and computational simulation, with support by S.J.P. Y.L., and D.K. supervised the study. S.‐R.L., Y.L., and D.K. secured the funding. All authors have approved the final version of the manuscript.

## Funding

This study was supported by the National Research Foundation of Korea (NRF) funded by the Ministry of Education, Science, and Technology (RS‐2022‐NR067413), and by the Korea Bio‐health Technology R&D Project through the Korea Health Industry Development Institute (KHIDI) funded by the Ministry of Health & Welfare (RS‐2023‐00267453). This study was supported by the Gachon University Research Fund of 2022 (GCU‐202209120001).

## Conflicts of Interest

The authors declare no competing financial interests or personal relationships that might have influenced the work reported in this study.

## Supporting information




**Supporting File**: advs76039‐sup‐0001‐SuppMat.docx.

## Data Availability

The data that support the findings of this study are available from the corresponding author upon reasonable request.
