# Supplementary material for: Surfaceome Reprogramming of Stemsomes Promotes Lung Cancer Targeting via Potentiated Receptor–Ligand Interactions
Source: Adv Sci (Weinh). 2026 Jun 9;13(51):e76039. doi: 10.1002/advs.76039 (PMC13336371; doi:10.1002/advs.76039)
Supplement: Supplementary file 1 [file ADVS-13-e76039-s001.docx]

Supporting Information

Surfaceome Reprogramming of Stemsomes Promotes Lung Cancer Targeting via Potentiated Receptor–Ligand Interactions

Geunhye Kim, In Ah Kwon, Bo Seop Jeong, Hyo Seon Kim, Joo-Hwan Park, Sung Jean Park, Sang-Rae Lee,* Youngki Lee,* and Dongwoo Khang*

Geunhye Kim, In Ah Kwon, Bo Seop Jeong, Dongwoo Khang

Department of Health Sciences and Technology, GAIHST, Gachon University, Incheon 21999, South Korea

Email: [dkhang@gachon.ac.kr](mailto:dkhang@gachon.ac.kr)

In Ah Kwon

Ectosome Inc., Incheon 21984, South Korea

Hyo Seon Kim, Dongwoo Khang

Department of Physiology, College of Medicine, Gachon University, Incheon 21999, South Korea

Joo-Hwan Park

Division of Medical Oncology, Department of Internal Medicine, Gachon University School of Medicine, Gil Medical Center, Incheon 21565, South Korea

Sung Jean Park

College of Pharmacy and Gachon Institute of Pharmaceutical Sciences, Gachon University, Incheon 21936, South Korea

Sang-Rae Lee

Department of Pharmacology, Ajou University School of Medicine, Suwon 16499, South Korea; Efficacy Test Center for Mental & Behavioral Disorders, Adjou University Hospital, Suwon 16499, South Korea

Email: [lsr21@ajou.ac.kr](mailto:lsr21@ajou.ac.kr)

Youngki Lee

Department of Biopharmaceutical Sciences, Cheongju University, Cheongju, 28160, South Korea.

Email: [brew071@cju.ac.kr](mailto:brew071@cju.ac.kr)

Youngki Lee, Dongwoo Khang

Lee Gil Ya Cancer and Diabetes Institute, Gachon University, Incheon 21999, South Korea

Geunhye Kim and In Ah Kwon contributed equally to this study.

**Table S1:** Nucleotide sequences of siRNAs used for gene silencing.

| **Target gene** | **Sequence** |
| --- | --- |
| *EPHA2* | 5'- CUC UAG UGC CUU CUU UAG Att-3' |
|  | 5'- UCU AAA GAA GGC ACU AGA Gtt-3' |
| *ITGA6* | 5'- CUC UAG GUA CGA UGA CAG Utt-3' |
|  | 5'- ACU GUC AUC GUA CCU AGA Gtt-3' |
| *ITGA3* | 5'- GAC AGU GAU GGG UGA GUC Utt-3' |
|  | 5'- AGA CUC ACC CAU CAC UGU Ctt-3' |
| *NOTCH3* | 5'- CAG UUC ACC UGU AUC UGU Att-3 |
|  | 5'- UAC AGA UAC AGG UGA ACU Gtt-3 |

**Table S2:** Primer sequences used for real-time PCR.

| **Target gene** | **Primer sequence** |
| --- | --- |
| *ITGA3* | 5'-GCC TGA CAA CAA GTG TGA GAG C-3' |
|  | 5'-GGT GTT CGT CAC GTT GAT GCT C-3' |
| *ITGA6* | 5'-CGA AAC CAA GGT TCT GAG CCC A-3' |
|  | 5'-CTT GGA TCT CCA CTG AGG CAG T-3' |
| *EPHA2* | 5'-CAC CAA GAC AGT TTG CTG CGG T-3' |
|  | 5'-GGA TGT TCA GGT TCT TGC CAG G-3' |
| *NOTCH3* | 5'-TAC TGG TAG CCA CTG TGA GCA G-3 |
|  | 5'-CAG TTA TCA CCA TTG TAG CCA GG-3 |
| *GAPDH* | 5'-AAG TAT GAC AAC AGC CTC AAG-3' |
|  | 5'-ATG AGT CCT TCC ACG ATA CCA-3' |

**Figure S1.** Optimization of dexamethasone preconditioning and stemsome-based nanocarrier preparation. (a) Quantification of mesenchymal stem cells (MSCs) migration toward H1975 cells after dexamethasone preconditioning at the indicated concentrations. Data are presented as mean ± SD (*n =* 9). ***P* < 0.01 compared to other groups except 100 nM. (b) Cell viability of MSCs in relation to dexamethasone concentration. Data are presented as mean ± SD (*n =* 8). (c) Sodium dodecyl sulfate-polyacrylamide gel electrophoresis (SDS-PAGE) showing total protein profiles (n = 1). (d) Protein expression level of CD44 using western blot (n = 1) and (e) flow cytometry (n = 1). (f) Drug content and encapsulation efficiency of each formulation. Data are presented as mean ± SD (*n =* 3). (g) Time-dependent changes in hydrodynamic size (n = 3). (h) Time-dependent viability of H1975 cells after drug treatment. Data are presented as mean ± SD (*n =* 10). ****P* < 0.001**,** *****P* < 0.0001 compared to CTL. (i) Cell viability of vehicle-only treatments (n = 3). CTL, control; Epi, epirubicin; HPF, high-power field.

 **Figure S2. Evaluation of the negative control and Body weight changes in mice.** (a) Representative image of a tumor free mice used for baseline signal extraction. (b) Body weight changes in mice. Data are presented as mean ± SD (n = 5). *****P* < 0.0001 for Cisplatin compared to CTL.

**Figure S3.** Transcriptomic profiling of naïve MSCs and D-MSCs. (a) Violin plots illustrating the distribution of log2-transformed gene expression levels in naïve MSCs and D-MSCs before and after filtering and normalization of mRNA-seq data. (b) Principal component analysis of normalized RNA-seq data showing the transcriptional separation between the distinct naïve MSCs and D-MSCs. The percentages indicate the proportion of variance explained by each principal component, PC1 and PC2. (c) Heatmap illustrating the hierarchical clustering of differentially expressed genes in naïve MSCs and D-MSCs. Expression values are shown as row-wise scaled Z-scores. (d) GSEA running enrichment score plots for gene sets related to cell–cell junction, molecular binding, localization to membrane, integrin complex, cadherin binding, and laminin binding. Enrichment scores were calculated based on ranked gene expression differences between naïve MSCs and D-MSCs.

**Figure S4.** Protein expression analysis in naïve MSCs and D-MSCs. (a) Western blot analysis of NOTCH3 expression. β-actin was used as a loading control (n = 1). (b) Flow cytometry analysis of EPHA2, ITGA3 and ITGA6 surface expression. Data are presented as mean ± SD (n = 3). ****P*< 0.001, ***P*<0.01.

**Figure S5.** Potential binding partners of D-MSCs and H1975 cells. Dot plot of potential PPIs between upregulated surface proteins on D-MSCs against their potential binding partners on H1975 cells. |logFC| > 1 and adjusted *P*-value < 0.05 for D-MSC. FC, fold change.

**Figure S6.** Binding interfaces of NOTCH3 and EPHA2 complexes. Visualization of the binding interfaces and interaction diagrams for (a) NOTCH3–JAG1 and NOTCH3–JAG2 and (b) EPHA2–EFNA1 and EPHA2–EFNB1.

**Figure S7.** Uncropped original gel and western blot images. (a) Total protein expression visualized by SDS-PAGE and subsequent Coomassie brilliant blue staining. (b) Original western blot images for CD44 and Na⁺/K⁺-ATPase (c) Original western blot images of NOTCH3 and β-actin in MSC and D-MSC. The inserted table provides quantitative analysis of band intensities.
